# A novel *PKD1* variant demonstrates a disease-modifying role in *trans* with a truncating *PKD1* mutation in patients with Autosomal Dominant Polycystic Kidney Disease

**DOI:** 10.1186/s12882-015-0015-7

**Published:** 2015-03-01

**Authors:** Hamad Ali, Naser Hussain, Medhat Naim, Mohamed Zayed, Fahd Al-Mulla, Elijah O Kehinde, Lauren M Seaburg, Jamie L Sundsbak, Peter C Harris

**Affiliations:** Department of Medical Laboratory Sciences, Faculty of Allied Health Sciences, Health Sciences Center, Kuwait University, Jabriya, Kuwait; Division of Nephrology, Mubarak Al-Kabeer Hospital, Ministry of Health, Jabriya, Kuwait; Department of Radio diagnosis, Mubarak Al-Kabeer Hospital, Ministry of Health, Jabriya, Kuwait; Department of Pathology, Faculty of Medicine, Health Sciences Center, Kuwait University, Jabriya, Kuwait; Department of Surgery, Division of Urology, Faculty of Medicine, Health Sciences Center, Kuwait University, Jabriya, Kuwait; Division of Nephrology and Hypertension, Mayo Clinic, Rochester, USA

**Keywords:** Polycystic kidney disease, ADPKD, PKD1, ESRD, Novel variant, Genetic modifier, eGFR, PKD

## Abstract

**Background:**

Autosomal Dominant Polycystic Kidney Disease (ADPKD) is the most common form of Polycystic Kidney Disease (PKD) and occurs at a frequency of 1/800 to 1/1000 affecting all ethnic groups worldwide. ADPKD shows significant intrafamilial phenotypic variability in the rate of disease progression and extra-renal manifestations, which suggests the involvement of heritable modifier genes. Here we show that the PKD1 gene can act as a disease causing and a disease modifier gene in ADPKD patients.

**Methods:**

Clinical evaluation of a family with ADPKD was performed to diagnose and assess disease progression in each individual. *PKD1* was genotyped in each individual by targeted sequencing.

**Results:**

Targeted screening analysis showed that the patients with ADPKD in the family had the *PKD1*: p.Q2243X nonsense mutation. A more severe disease phenotype, in terms of estimated Glomerular Filtration Rate (eGFR) and total kidney volume, was observed in two patients where in addition to the mutation, they carried a novel *PKD1* variant (p.H1769Y). Other patients from the same family carrying only the (p.Q2243X) mutation showed milder disease manifestations.

**Conclusion:**

ADPKD shows significant intrafamilial phenotypic variability that is generally attributed to other modifier genes. In this rare case, we have shown that a variant at *PKD1, in trans* with the *PKD1* mutation, can also act as a modifier gene in ADPKD patients. Understanding the molecular mechanism through which the gene exerts its disease modifying role may aid our understanding of the pathogenesis of ADPKD.

## Background

Autosomal dominant polycystic kidney disease (ADPKD) is the most common renal hereditary disease affecting one in every 800–1000 individuals worldwide [1]. It is characterized mainly by growth of bilateral multiple renal cysts and expansion of kidney size, in an age related manner, leading to progressive impairment of kidney functions, which ultimately results in end-stage renal disease (ESRD). ADPKD patients can also develop extrarenal manifestations including hepatic and pancreatic cysts, cerebral and aortic aneurysms, cardiac valvular abnormalities and systematic hypertension [1-3].

ADPKD is genetically heterogeneous as it is caused by mutations in either of two genes: *PKD1,* which is located on chromosome 16 (49,511 bp with 46 exons) and *PKD2,* which is located on chromosome 4 (70,133 bp with 15 exons). *PKD1* mutations account for around 85% of the ADPKD cases in clinically identified populations, while mutations in *PKD2* accounted for the remaining 15% [4,5]. Phenotypes associated with ADPKD in terms of age of onset of ESRD, associated liver disease and other extrarenal manifestations show high levels of variability between patients, reviewed in [2,6]. This phenotypic variability can be attributed to genic and allelic heterogeneity. In general, mutations in *PKD1* are associated with more severe disease and earlier mean of age at onset of ESRD than mutations in *PKD2* (54.3 years for *PKD1* and 74 years for *PKD2*) [7-9]. At the allelic level, certain mutations are associated with more severe disease phenotype than others [10]. For example, on average, patients with truncating mutations have a more severe disease phenotype than patients with non-truncating mutations [7].

Several studies have suggested that functional gene dosage plays a determining role in ADPKD manifestation and severity. It has been shown that homozygous inheritance of incompletely penetrant *PKD1* alleles can be associated with typical ADPKD manifestation and ESRD, while heterozygous inheritance of the same alleles was associated with a mild cystic disease. Moreover, the inheritance of an incompletely penetrant *PKD1* allele *in trans* with *PKD1* inactivating mutation has been associated with early onset ADPKD. These cases suggest that dosage of the functional PKD1 protein (Polycystin-1) influences disease onset and can contribute to the phenotypic variability observed in cases of ADPKD [11-13].

Another element that adds to the complexity of phenotypic variability in ADPKD is the involvement of modifier genes that are suggested by the intrafamilial phenotypic variability observed in ADPKD families where patients share the same mutation but yet show significant differences in disease severity and presentation [14,15]. Several studies have highlighted a possible modifying role for a number of genes in ADPKD patients including *ENOS* and *ACE.* However, this role is debatable as a number of other studies showed these genes have no significant role in the disease progression, severity and phenotypic variability [16-20]. It was also shown that mutations in other PKD genes like *PKHD1* and *HNF1B* when co-inherited with *PKD1* or *PKD2* mutation can cause early onset of PKD [11]. Other studies suggested phenotypic modifying roles for *TSC2* and *DKK3* [21,22]. Identifying modifier genes that are responsible for the substantial clinical variability observed in ADPKD across families would allow better prediction of disease prognosis and contribute to better management prior to onset of ESRD. It would also allow better understanding of the molecular pathways involved in the disease pathology which is important for the development of potential therapies.

Here, we show that *PKD1* is acting as a disease causing and disease-modifying gene. We show that a novel *PKD1* variant demonstrates a disease-modifying role in *trans* with a *PKD1* disease causing mutation in a family with ADPKD.

## Methods

### Inclusion criteria

Families with history of ADPKD were selected for the study when individuals showed typical clinical presentation of ADPKD including multiple renal cysts and reduced kidney functions. The study was approved by the joint committee for the protection of human subjects in research of the Health Sciences Center (HSC) and Kuwait Institute for Medical specialization (KIMS) (Reference: VDR/JC/690). Written informed consent was obtained from all patients prior to involvement in the study according to the laws and regulations of the joint HSC and KIMS ethical committee. The pedigree of the family was drawn using the Progeny drawing tool.

### DNA isolation

A 10 ml blood sample was collected from each patient by a qualified nurse at the nephrology department in Mubarak Al-Kabir Hospital in Kuwait and processed immediately. Genomic DNA was isolated from peripheral blood using Gentra Puregene Blood Kit (Qiagen, 158467) following the manufacturer’s protocol.

### Mutation screening and classification of variants

Mutations were screened in the proband of the family by locus specific amplification of *PKD1* and direct sequencing of exonic and flanking intronic regions of *PKD1* and *PKD2* [4]. Segregation was tested by sequence analysis of the relevant genomic fragments in family members. The significance of missense variants was assessed using the ADPKD Mutation Database http://pkdb.mayo.edu, multi-sequence alignments and substitution assessment tools: SIFT, PolyPhen2 and Align GVGD, as previously described [12,13].

### Clinical evaluation

Clinical evaluation was performed on individuals at risk, where at least a parent or a sibling showed typical ADPKD manifestations. Individuals who showed negative results in mutation screening were also clinically evaluated to confirm their disease status, clinical results confirmed mutation screening. Results not shown.

### Abdominal ultrasound

All individuals except those with kidney transplants were instructed to fast 4–6 hours prior to abdominal ultrasound examinations which was performed on using logic 7 GE machine with curvilinear 3.5 MHZ probe. Multiple positions were used to assess the abdominal structures, initially supine and then lying on both sides. Focused ultrasound was performed to assess both kidneys, and the liver and pancreas. Initially each kidney was assessed in multiple views. The presence or absence of cysts was examined and the exact number recorded when the cyst number was equal or less than 20 in each kidney. If the cyst number exceeded 20 in each kidney it was recorded as >20. Each kidney volume was calculated following the formula: ([Antero-posterior X Bipolar X Side-side diameters]/2) automatically by the machine and expressed in cubic centimeters (cc). Total kidney volume was calculated and height-adjusted total kidney volume (htTKV) expressed in cubic centimeters per meter (cc/m). Liver and pancreas were also screened for presence or absence of cysts.

### Renal function test

A 5 ml blood sample was taken from all individuals for Renal Function Test (RFT). The test was performed in the main laboratory at Mubarak Al-Kabir Hospital in Kuwait. Serum Creatinine levels were determined for each patient in μmol/l. Another 5 ml blood sample was taken from all individuals to determine serum Cystatin C levels using Human Cystatin C Quantikine ELISA Kit (DSCTC0, R&D systems). The test was performed according to manufacturer’s protocol in the laboratories of the MLS Department in Faculty of Allied Health Sciences in Kuwait University. Blood samples were centrifuged at 2000 rpm for 10 minutes to isolate the serum. 100ul of assay diluent was added to each well and then 50 ul of standard, control, and sample were added to each well and then covered and incubated for 3 hours at 2–8°C. Wells were aspirated and procedure repeated for a total of four washes. 200ul of cold Cystatin C conjugate was added to each well, and incubated for 1 hour at 2–8°C. Wells were aspirated and washed 4 times, 200ul of substrate solution was added to each well and incubated for 30 minutes at room temperature for one hour. Stop Solution (50 μL) was added to each well and the optical density was determined using a microplate reader set to 450 nm.

Estimated Glomerular Filtration Rates (eGFR) was calculated using the CKD-EPI CREATININE-CYSTATIN C (2012) equation developed by Inker et al. [23]. Calculations were performed using the GFR calculator provided on The National Kidney Foundation website www.kidney.org.

## Results

### Genetic analysis

**Figure 1:**
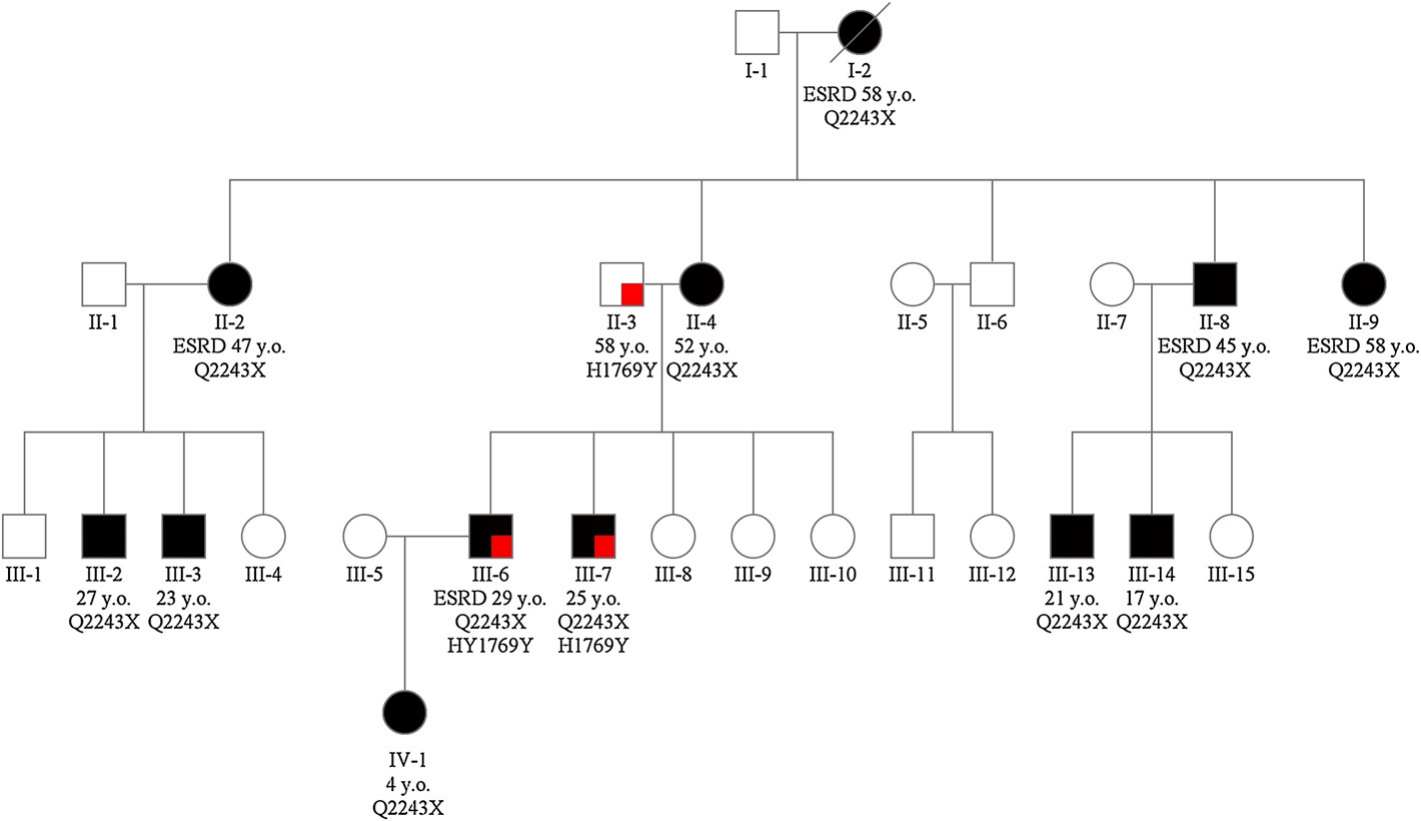
**Pedigree of the ADPKD family showing the PKD1 genotype of each member along with age and onset of ESRD.**

**Figure 2:**
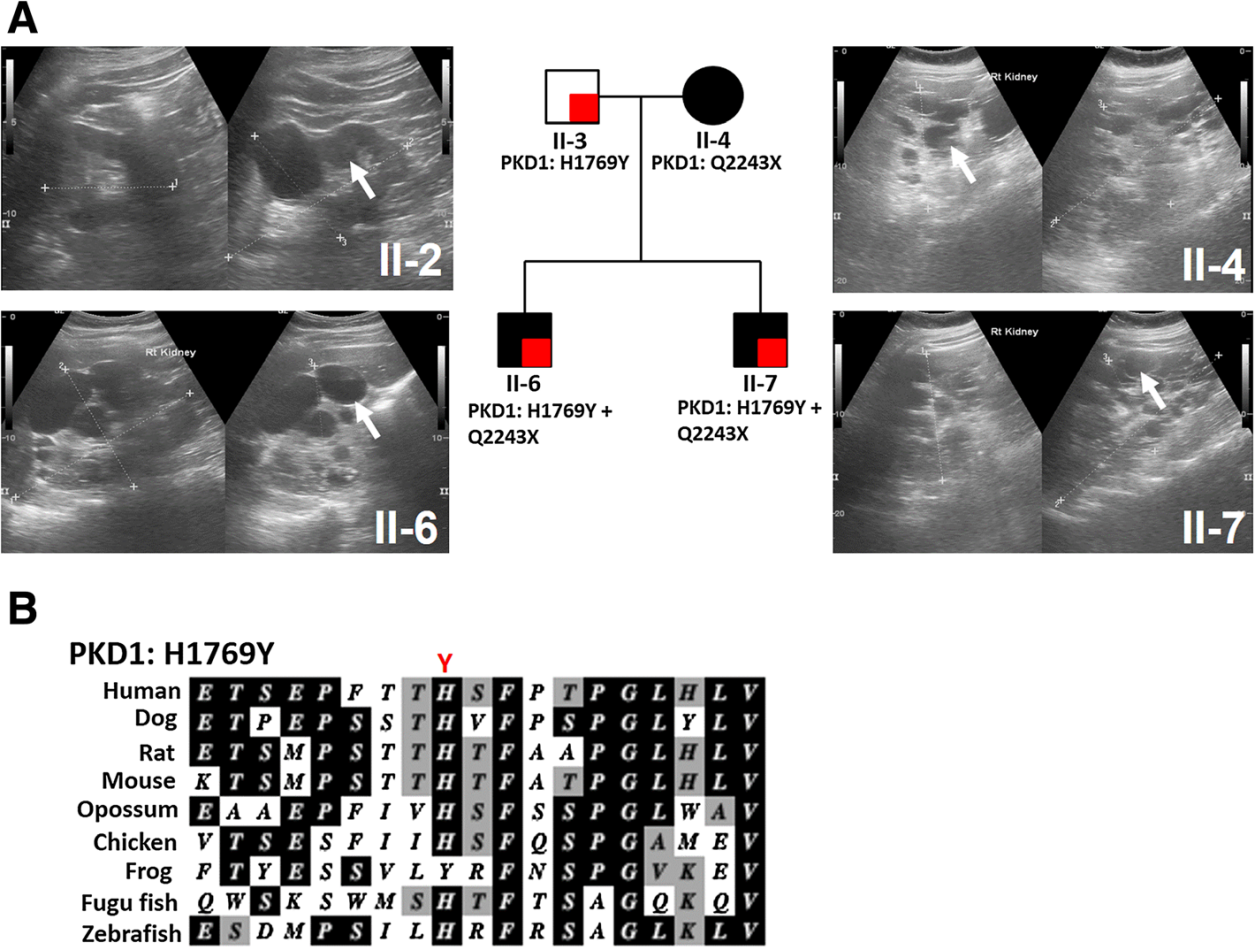
**Inheritance of** ***PKD1*** **novel variant (p.H1769Y) and mutation (p.Q2243X). (A)** Renal ultrasound analysis of patients inheriting the *PKD1* novel variant (p.H1769Y) and mutation (p.Q2243X). Patient (II-3) had normal kidney size with a total of 7 renal cysts. Patient (II-4) showed extremely enlarged kidneys with multiple renal cysts and a GFR of 25 mL/min/1.73 m^2^ at the age of 52. Patients (II-6) showed extremely enlarged kidneys with multiple renal cysts and reached renal failure at the age of 29. Patient (II-7) showed extremely enlarged kidneys with multiple cysts at the age of 25. White arrows showing large cysts **(B)** Multi sequence alignment of polycystin-1 orthologs showing conservation of p.H1769.

The family pedigree demonstrates the typical pattern of inheritance seen in families with ADPKD (Figure 1). Twenty-eight family members were enrolled in the study representing four generations. Locus-specific PCR and Sanger sequencing revealed that a *PKD1* mutation (p.Q2243X) segregates with the disease in the studied family that generally showed typical ADPKD manifestations. p.Q2243X (c.6727C > T) is a known nonsense mutation located in exon 15 of *PKD1*. Individuals II-3, III-6, III-7 and IV-2 carried a novel *PKD1* variant (p.H1769Y; c.5305C > T; exon 15). Histidine 1769 is invariantly conserved in orthologs of the PKD1 protein, polycystin-1, to fish, except in frog where it matches the substitution to tyrosine (Figure 2B). Histidine 1769 is found within PKD Repeat XII and histidine is the most commonly found residue at this position in other PKD Repeats. Altogether we scored the variant as of Indeterminate significance [4]. Individuals III-6 and III-7 carried both the mutation (p.Q2243X) and the novel variant (p.H1769Y) (Figure 1).

The variant effect was evaluated using prediction tools including Polyphen-2, SIFT and Mutation taster (Table 1). Scores from Polyphen-2 and SIFT suggested that the variant has a benign effect while the Grantham Matrix Score suggested a possible impact on protein features and splice site changes.

**Table 1:** **Novel PKD1 variant effect prediction analysis**

**Gene**	**Variant DNA**	**Variant protein**	**Genotype**	**Conserved (Yes/No)**	**Polyphen-2 Score** ^**a**^	**SIFT score** ^**b**^	**The Grantham Matrix score** ^**c**^	**Novel (Yes/No)**
*PKD1*	c.5305C > T	p.H1769Y	Heterozygous	Yes	0.022	0.25	83	Yes

^a^Score range 0–1.0, 1.0 is more damaging.

^b^Score range from 0 to 1.0. Scores ≤0.05 indicate damaging effect , scores >0.05 indicate tolerated effect.

^c^Score range 0–215. Higher score predicts more significant impact. The Grantham Matrix Score predicts the impact of amino acid substitution on the protein structure and function.

### Clinical evaluation

Among the 28 family members, the 12 individuals that carried the PKD1 mutation (p.Q2243X) showed typical ADPKD manifestations including renal cysts, and renal enlargement in all but the one case where the data was unavailable (Table 2). Five patients had already reached ESRD at the time the study was performed, and one other had a significant decline in eGFR. Patient (III-6), who carries both the mutation and the novel variant (p.H1769Y), showed more severe disease than the rest of the family as he reached ESRD at the age of 29 years old. He inherited the mutation from his mother (II-4) who is 52 years old and with Chronic Kidney Disease (CKD) stage 4. The average onset of ESRD of the patients carrying the mutation alone was 52 years old.

**Table 2:** **Clinical and genetic evaluation of family members**

**Patient code**	**Age on the day (years)**	**Gender**	**Number of cysts**			**GFR (mL/min/1.73 m2)**	**ESRD onset (years)**	**htTKV (cc/m)**	**Renal transplant**	**Mutation**	**Variant**
			**Kidneys**	**Liver**	**Pancreas**						
I-2	Deceased	F	>40	0	0	-	58	-	Yes	Q2243X	-
II-2	57	F	>40	0	0	-	47	-	Yes	Q2243X	-
II-3	58	M	7	0	0	94	-	312.5	No	-	H1769Y
II-4	52	F	>40	0	0	25	-	1742.1	No	Q2243X	-
II-8	56	M	>40	0	0	-	45	-	Yes	Q2243X	-
II-9	58	F	>40	0	0	-	58	-	Yes	Q2243X	-
III-2	27	M	>40	0	0	85	-	865.4	No	Q2243X	-
III-3	23	M	11	0	0	109	-	359.2	No	Q2243X	-
III-6	30	M	>40	0	0	6	29	1362.6	No	Q2243X	H1769Y
III-7	25	M	>40	0	2	91	-	1344.1	No	Q2243X	H1769Y
III-13	21	M	23	0	0	109	-	491.7	No	Q2243X	-
III-14	17	M	>40	0	0	112	-	597.7	No	Q2243X	-
IV-1	4	F	2	0	0	130	-	130	No	Q2243X	-

**Figure 3:**
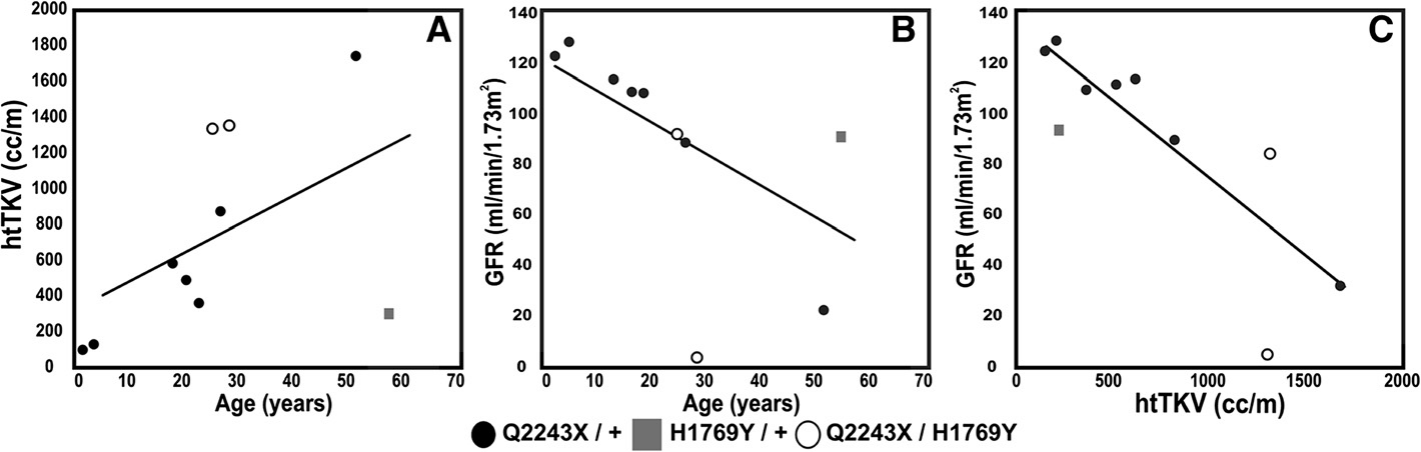
**Correlation between height-adjusted total kidney volume (htTKV) or eGFR and age, (A) and (B), plus correlation of htTKV and eGFR (C).** The patients carrying the mutation and novel variant had larger kidney volume than patients of the same age carrying the mutation, while one of the patients with both variants had ESRD. The patient with just p.H1769Y had normal sized kidneys and normal eGFR.

In the family, the two patients (III-6 and III-7) carrying both the mutation (p.Q2243X) and the novel variant (p.H1769Y) had larger kidneys than other patients of similar age (Table 2, Figures 2 and 3A). Patient (III-6) had htTKV of 1362.6 cc/m at the time of ESRD (29 years) while patient (III-7) had htTKV of 1344.1 cc/m by the age of 25, although his renal function was in the normal range (Figure 3B). Renal ultrasound analysis of patient II-4 showed numerous cysts of different sizes with the largest detected measuring 5.5 cm X 4 cm on the left kidney at its lower pole, all with thin walls and no septation (Figure 2A). Patient III-6 showed numerous cysts of varying sizes, some of which had fine internal septation. Patient III-7 showed numerous renal cysts with various sizes with the largest detected on right kidney measuring 2.6 cm X 2.4 cm. Interestingly, individual (II-3) who harbors only the novel variant showed normal kidney volume and function but had a total of 7 renal cysts at the age of 58 (Table 2 and Figure 3A and B). The left kidney had 2 small cysts with less than 1 cm at maximum diameters and one large midpolar cyst measuring 4.5 cm X 4.25 cm. The right kidney had 4 cysts, one is relatively large of about 3 cm X 2.4 cm while the rest are small with less than 1 cm at maximum diameters. All cysts noted are of thin wall with no internal septation (Figure 2A).

## Discussion

ADPKD shows a high degree of phenotypic variability between patients due to genic and allelic heterogeneity, and environmental influences. Involvement of modifier genes, suggested by intrafamilial phenotypic variability, adds more complexity to the genotype-phenotype correlation in ADPKD [2]. Identifying those modifier genes and understanding how they influence disease phenotype not only will improve disease prognosis and management but it will also aid the development of potential therapies.

In this study, we identified a novel *PKD1* variant (p.H1769Y) that demonstrated a disease-modifying role, *in trans* with the *PKD1* truncating mutation, in ADPKD patients. The variant is at a well-conserved histidine, which is replaced by tyrosine, a non-conservative change, which suggest that this position is important for proper protein function (Figure 2B). To evaluate the effect of the novel variant on the disease severity and progression, patients were clinically evaluated using ultrasound to examine cysts and total kidney volume and by measuring GFR using creatinine and cystatin C serum levels. Overall, our results showed that renal volume expansion was associated with declined renal functions and older age in the examined ADPKD family, a pattern normally seen in families with ADPKD (Figure 3) [24-26].

To evaluate the effect of the novel variant on kidney functions, we first examined the individual who carries only the (p.H1769Y) variant, a 58 years old male (II-3). Ultrasound examination revealed that patient (II-3) had normal kidney volume with a total of seven renal cysts and his renal function was normal by eGFR (Figure 3). Finding seven cysts by ultrasound in an individual of 58 years would be unusual and meet the criteria for a diagnosis of ADPKD if they had a positive family history [27]. In this case as they do not have a family history of ADPKD, generally the more stringent criteria of 10 cysts per kidney would be applied [28], but despite not meeting that threshold the finding of seven cysts suggests that p.H1769Y is of some phenotypic significance.

To further evaluate the possible disease modifying role of the novel variant, we clinically evaluated and compared the disease progression in patients carrying both the mutation (p.Q2243X) and the novel variant (p.H1769Y) to patients carrying only the mutation. Patient (III-6) reached ESRD at the age of 29, 23 years earlier than average ESRD onset in the family indicating a more severe disease progression. Additionally, his kidneys were extremely enlarged (htTKV 1362.6 cc/m) in comparison with patients in the same age group carrying only the mutation (III-2 and III-3) (Table 2 and Figure 3A). The other patient carrying both the variant and mutation (III-7) although showing normal kidney functions at the age of 25 (eGFR 91 mL/min/1.73 m2), his kidneys were also extremely enlarged (htTKV 1344.1 cc/m) in comparison to patients in the same age group carrying only the (p.Q2243X) mutation (III-2 and III-3) (Table 2 and Figure 3A).

Serum creatinine and cystatin C levels are elevated at late stages of the PKD and therefore GFR does not represent a sensitive tool to monitor the disease progression. However, kidney volume enlargement reflecting cysts development and growth appears to be a continuous process throughout the course of the disease and therefore has been proposed as an indicator to disease severity and progression even before GFR changes can be detected [24-26]. This suggests severe disease in patient (III-7) despite stable GFR at the age of 25.

Variant effect prediction tools like Polyphen-2 and SIFT scored the variant as benign. While Grantham Matrix score suggested that the variant could have an effect on protein features and splice sites. These findings agree with our clinical findings where the novel variant, on its own, is not ADPKD causing, as observed in individual (II-3). However when the variant is co-inherited with a pathogenic mutation it worsen the disease phenotype which suggest a possible molecular effect on protein features as suggested by the Grantham Matrix Score (Table 1). These observations suggest that the effect of this variant could be tolerated when co-inherited with healthy *PKD1,* following recessive mode of inherence, but when co-inherited with a disease causing mutation, it acts as a disease modifier rather not disease causing variant. At the molecular level, the substitution of histidine to tyrosine could affect the overall stability and functionality of the proteins as these two amino acids show some different chemical properties in the form of positively charged side chain in histidine but not in tyrosine.

Around 2% of ADPKD patients show an early and severe disease phenotype. These cases can be caused by co-inheritance of two *in trans PKD1* mutations resulting in severe and early disease phenotype [12], or by coinheritance of a mutation in another cystogene [11]. These cases sometimes can be indistinguishable clinically from the recessive form of the disease [13]. In our case, we propose that the novel variant (p.H1769Y) aggravated the disease phenotype in patients (III-6 and III-7), resulting in early onset of ESRD and renal enlargement.

## Conclusion

In summary, we propose that the novel *PKD1* variant has a disease-modifying role in *trans* with the *PKD1* mutation in the studied family. Further molecular analysis is required to investigate the molecular effect the single amino acid substitution has on the polycystin-1 protein. Understanding the molecular pathological basis of the modifying role of the variant (p.H1769Y) would provide insights to potential therapies for the disease.

## Abbreviations

ADPKD: Autosomal dominant polycystic kidney disease
eGFR: Estimated glomerular filtration rates
ESRD: End-stage renal disease
htTKV: Height-adjusted total kidney volume
PKD: Polycystic kidney disease
